# Fully automated AI-based splenic segmentation for predicting survival and estimating the risk of hepatic decompensation in TACE patients with HCC

**DOI:** 10.1007/s00330-022-08737-z

**Published:** 2022-04-08

**Authors:** Lukas Müller, Roman Kloeckner, Aline Mähringer-Kunz, Fabian Stoehr, Christoph Düber, Gordon Arnhold, Simon Johannes Gairing, Friedrich Foerster, Arndt Weinmann, Peter Robert Galle, Jens Mittler, Daniel Pinto dos Santos, Felix Hahn

**Affiliations:** 1 Department of Diagnostic and Interventional Radiology, University Medical Center of the Johannes Gutenberg University Mainz, Langenbeckst. 1, 55131 Mainz, Germany; 2 Department of Internal Medicine I, University Medical Center of the Johannes Gutenberg University Mainz, Mainz, Germany; 3 Department of General, Visceral and Transplant Surgery, University Medical Center of the Johannes Gutenberg University Mainz, Mainz, Germany; 4 Department of Radiology, University Hospital of Cologne, Cologne, Germany; 5 Institute for Diagnostic and Interventional Radiology, Goethe-University Frankfurt am Main, Frankfurt, Germany

**Keywords:** Hepatocellular carcinoma, Transarterial chemoembolization, Artificial intelligence, Splenic volume

## Abstract

**Objectives:**

Splenic volume (SV) was proposed as a relevant prognostic factor for patients with hepatocellular carcinoma (HCC). We trained a deep-learning algorithm to fully automatically assess SV based on computed tomography (CT) scans. Then, we investigated SV as a prognostic factor for patients with HCC undergoing transarterial chemoembolization (TACE).

**Methods:**

This retrospective study included 327 treatment-naïve patients with HCC undergoing initial TACE at our tertiary care center between 2010 and 2020. A convolutional neural network was trained and validated on the first 100 consecutive cases for spleen segmentation. Then, we used the algorithm to evaluate SV in all 327 patients. Subsequently, we evaluated correlations between SV and survival as well as the risk of hepatic decompensation during TACE.

**Results:**

The algorithm showed Sørensen Dice Scores of 0.96 during both training and validation. In the remaining 227 patients assessed with the algorithm, spleen segmentation was visually approved in 223 patients (98.2%) and failed in four patients (1.8%), which required manual re-assessments. Mean SV was 551 ml. Survival was significantly lower in patients with high SV (10.9 months), compared to low SV (22.0 months, *p* = 0.001). In contrast, overall survival was not significantly predicted by axial and craniocaudal spleen diameter. Furthermore, patients with a hepatic decompensation after TACE had significantly higher SV (*p* < 0.001).

**Conclusion:**

Automated SV assessments showed superior survival predictions in patients with HCC undergoing TACE compared to two-dimensional spleen size estimates and identified patients at risk of hepatic decompensation. Thus, SV could serve as an automatically available, currently underappreciated imaging biomarker.

**Key Points:**

*• Splenic volume is a relevant prognostic factor for prediction of survival in patients with HCC undergoing TACE, and should be preferred over two-dimensional surrogates for splenic size*.

*• Besides overall survival, progression-free survival and hepatic decompensation were significantly associated with splenic volume, making splenic volume a currently underappreciated prognostic factor prior to TACE*.

*• Splenic volume can be fully automatically assessed using deep-learning methods; thus, it is a promising imaging biomarker easily integrable into daily radiological routine*.

**Supplementary Information:**

The online version contains supplementary material available at 10.1007/s00330-022-08737-z.

## Introduction

Hepatocellular carcinoma (HCC) is the most common primary liver cancer worldwide, and it ranks second among diseases responsible for cancer-related deaths [1, 2]. More than 80% of HCCs develop as a consequence of liver cirrhosis [3]. Thus, most patients with HCC have two different diseases: HCC and liver cirrhosis. Hence, it is essential to assess both tumor burden and remnant liver function in making optimal treatment decisions [3, 4].

In addition to compromising liver protein synthesis, cirrhosis also leads to progressive changes in the splanchnic circulation [5]. During cirrhosis, continuous tissue re-organizations lead to an increase in portal pressure. Portal hypertension ultimately leads to the development of gastroesophageal varices, ascites, and splenic volume increases [6, 7]. Consequently, a high splenic volume is related to severe liver cirrhosis [8]. Accordingly, the splenic volume has been identified as a highly sensitive prognostic parameter for patients with HCC undergoing resection or tumor ablation [9–11]. In an initial study in patients with HCC that underwent transarterial chemoembolization (TACE), splenic volume was recently identified as a relevant prognostic factor [12]. Progression-free survival and hepatic decompensation were not investigated. Moreover, the sample size was small and spleen volume was assessed manually [12]. Manual splenic volume assessments on cross-sectional computed tomography (CT) images is a time-consuming task with a high risk of interrater variance [13]. Thus, it is not feasible in daily clinical routine.

Fortunately, recent developments in the field of artificial intelligence, particularly deep learning, have provided knowledge about automated organ segmentation and volume assessments. These automated algorithms can be readily integrated into clinical workflows in real time [14]. Hence, splenic volume might become an easily assessable and readily available prognostic factor for treatment planning and post-TACE follow-ups.

This study had two primary research goals: First, we aimed to build a deep-learning algorithm for fully automated splenic volume assessments based on CT images. Second, we aimed to validate the role of total splenic volume as a novel imaging biomarker for survival prediction and to investigate its role as an indicator for hepatic decompensation in patients with HCC undergoing TACE.

## Methods

The Ethics Committee of the Medical Association of Rhineland Palatinate, Mainz, Germany, approved this study (permit number 2021-15984). The requirement for informed consent was waived, due to the retrospective nature of the study. Patient records and information were anonymized prior to analysis. This report followed the guidelines for transparent reporting of a multivariable prediction model for individual prognosis or diagnosis (TRIPOD) and the guidelines for reporting observational studies (STROBE) (Supplementary Tables 1 and 2) [15, 16].

### Patients

**Fig. 1:**
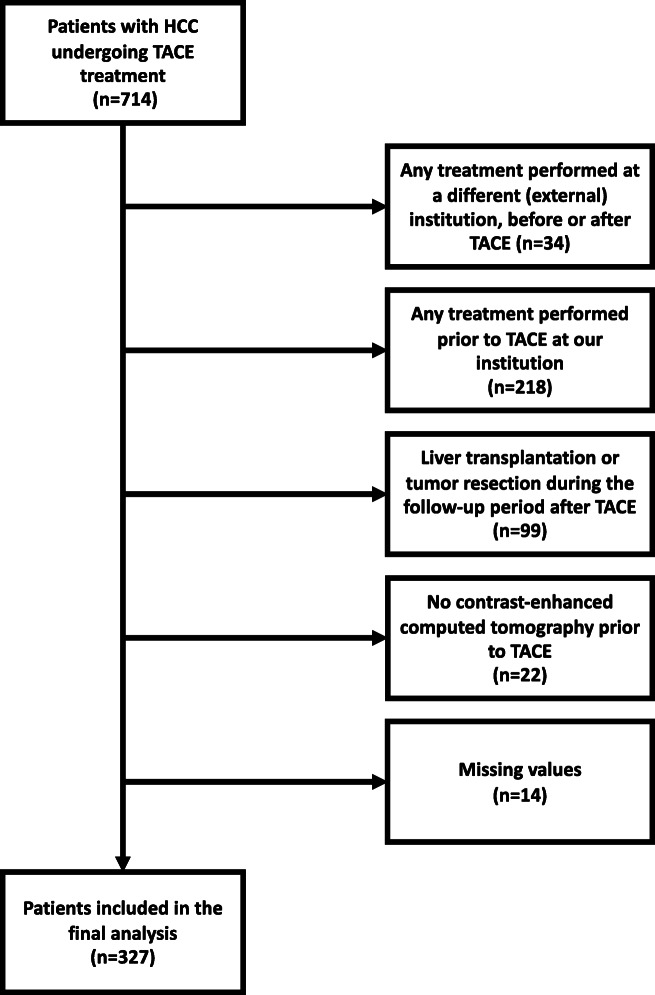
Flowchart of the patient selection process for this study

We identified 714 patients with confirmed HCC that received TACE treatment in our tertiary care center between January 2010 and November 2020. Of these, 327 patients fulfilled the following inclusion criteria: (1) age above 18 years; (2) histologically or image-derived HCC diagnosis based on the EASL criteria; (3) no treatment performed prior to TACE; (4) no liver transplantation or tumor resection during the follow-up period after TACE; (5) pre-interventional contrast-enhanced CT scan for splenic volume assessment; (6) full availability of clinical, laboratory, and imaging data. A total of 387 patients were excluded, due to reasons shown in Fig. 1.

### Diagnosis, treatment, and follow-up

HCC was diagnosed, based on histological or image-derived criteria established by the European Association for the Study of the Liver, as previously described [3, 17]. All patients underwent contrast-enhanced CT for treatment planning. Indications for TACE were discussed in an interdisciplinary tumor board, which included hepatologists/oncologists, diagnostic and interventional radiologists, visceral surgeons, pathologists, and radiation therapists. TACE was performed in a standardized manner, as previously described [18, 19]. Follow-ups included cross-sectional imaging, a clinical examination, and blood sampling. Follow-ups were performed every 6 or 12 weeks, depending on the presence of viable tumor tissue [17]. Radiologic response was assessed using mRECIST criteria [3, 20]. The first primary endpoint was the median overall survival (OS), defined as the interval between the initial TACE session and the date of death or last follow-up. Moreover, we investigated hepatic decompensation after TACE, which was objectively defined as an increase of the ALBI grade 3 months after the initial TACE as previously proposed [21, 22]. Further endpoints were the time to progression (TTP) and the time to untreatable (unTACEable) progression (TTUP). UnTACEable progression is defined as a clinical status that prohibits further TACE [3]. This status is caused by non-response of target lesions after two or more TACE sessions, new extrahepatic tumor spread, or hepatic decompensation [23].

### Data acquisition

The dataset was acquired from the clinical registry unit, as previously reported [17]. This dedicated, prospectively populated database contained data on all patients with primary liver cancer [24]. Additional imaging and laboratory data were acquired from the radiology information system and the laboratory database. The final dataset included all available data on patient demographics, clinical assessments of the underlying liver disease and tumor, imaging, factors related to the TACE treatment, and laboratory parameters measured prior to the initial TACE treatment [17].

### Splenic volume assessment: algorithm design, training, and validation

We used the open source Python library MIScnn (https://github.com/frankkramer-lab/MIScnn) to train an automated algorithm. The library provides a convolutional neural network with a U-Net architecture, which allows the segmentation of 3D medical images [25]. Thus, we extracted the portal venous phase of the abdominal CT scans for all patients in our dataset. For the first 100 consecutive patients in the dataset, we manually segmented the spleen with the freely available LIFEx software (www.lifexsoft.org) [26]. However, as MIScnn reads NIfTI image segmentation files (https://nifti.nimh.nih.gov/), any segmentation software which is able to save in this format could have been used.

Next, we used the first 70 manually segmented spleens for training a spleen segmentation algorithm, and the remaining 30 manually segmented spleens to validate the neural network. Preprocessing of the images (resampling to 2 × 2 × 3 mm, clipping from −50 to 350 to Hounsfield units, and *Z*-score normalization) and six cycles of data augmentation in the training set were performed as provided by the library. During training, the Tversky loss was calculated and we implemented Keras callback functions to facilitate early stopping and to reduce the learning rate on plateaus [27, 28]. Training was performed for 300 epochs. For postprocessing, we selected the largest connected region in the left half of the abdomen to avoid rare cases of erroneous partial liver segmentation.

After training, the portal venous phase CT scans of the remaining 227 patients were automatically processed by the algorithm without the need of further human preprocessing. We evaluated the results with graphic overlays of the predicted segmentation for these remaining 227 patients, and splenic volume was automatically calculated. The graphic overlays were used by two independent readers to rate the performance of the algorithm (i.e., perfect, acceptable, or poor). In cases of discrepancy, a consensus reading was performed. Patients with perfect or acceptable computed splenic segmentations were included in the statistical analyses; patients with a poor grade were assigned to manual segmentation for further analysis. Additionally, spleen volume in relation to the body surface area (BSA) was calculated, which was assessed using the patient’s height and weight.

Axial and craniocaudal spleen sizes were manually assessed in a standardized manner, as previously described [13].

### Statistical analysis

All statistical analyses and graphics were performed in R studio (RStudio Team [2020]. RStudio: Integrated Development for R. RStudio, PBC, http://www.rstudio.com, last accessed 30 Sept 2021) with R 4.0.3 (A Language and Environment for Statistical Computing, R Foundation for Statistical Computing, http://www.R-project.org; last accessed 10 Jan 2022). Binary and categorical baseline parameters are expressed as absolute numbers and percentages. Continuous data are expressed as the median and range. Subgroups were compared with the chi-square test and Mann-Whitney *U*-test. The Sørensen Dice Score was calculated to assess algorithm performance. Furthermore, manual and automated splenic volume assessments were compared with a Bland-Altmann plot. Survival analyses were performed with the packages “survminer” and “survival” (https://cran.r-project.org/package=survminer, https://CRAN.R-project.org/package=survival, last accessed 10 Jan 2022). Survival was evaluated with Kaplan–Meier curves, and strata were compared with log-rank testing. We used the same packages to determine cut-off values for splenic volumes, based on optimal stratification. We built univariate and multivariate Cox proportional hazards regression models and assessed hazard ratios (HRs) and the corresponding 95% confidence intervals (CIs). Sensitivity, specificity, and area under the curve (AUC) were calculated using the package “pROC” (https://cran.r-project.org/web/packages/pROC/index.html, last accessed 10 Jan 2022). The optimal cutoff for maximized sensitivity and specificity was determined using the Youden Index. *p*-values < 0.05 were considered statistically significant.

## Results

### Baseline

The baseline characteristics of patients with HCC at the initial TACE treatment are shown in Table 1.

**Table 1:** Baseline patient characteristics

Variable	All patients (*n* = 327)
Median age, years (IQR)	69.1 (62.6–75.4)
Sex, *n* (%)	
Female	51 (15.6)
Male	276 (84.4)
Etiology, *n* (%)^a^	
Alcohol	156
Hepatitis C	55
Hepatitis B	28
NAFLD	26
Hemochromatosis	9
AIH/PBC/PSC	5
Unknown/other	27
Child-Pugh stage, *n* (%)	
A	120 (36.7)
B	133 (40.7)
C	30 (9.2)
No cirrhosis	44 (13.4)
BCLC stage, *n* (%)	
0	0
A	60 (18.3)
B	166 (50.8)
C	71 (21.7)
D	30 (9.2)
Median tumor size, mm (IQR)	42 (28–64)
Tumor number, *n* (%)	
Unifocal	74 (22.6)
Multifocal	221 (67.6)
Diffuse growth pattern	32 (9.8)
Median albumin level, g/l (IQR)	31 (27–35)
Median bilirubin level, mg/dl (IQR)	1.4 (0.8–2.2)
Median platelet count, per nl (IQR)	129 (87–192)
Median AST level, U/l (IQR)	64 (46–100)
Median ALT level, U/l (IQR)	41 (28–61)
Median INR (IQR)	1.2 (1.1–1.3)
Median AFP level, ng/ml (IQR)	30 (7–767)
Number of TACE sessions, *n* (%)	
Single	84 (25.7)
Multiple	243 (74.3)
Subsequent treatment	
Yes^b^	72 (22.0)
No	255 (78.0)

^a^More than one etiology was possible for liver disease; thus, percentages were not calculated. Abbreviations: *NASH*, nonalcoholic steatohepatitis; *AIH*, autoimmune hepatitis; *PBC*, primary biliary cholangitis; *PSC*, primary sclerosing cholangitis; *BCLC*, Barcelona Clinic Liver Cancer; *AST*, aspartate aminotransferase; *ALT*, alanine aminotransferase; *AFP*, alpha fetoprotein. ^b^Sorafenib (*n* = 33), lenvatinib (*n* = 13), selective internal radiation therapy (*n* = 12), atezolizumab in combination with bevacizumab (*n* = 6), pembrolizumab (*n* = 2), pembrolizumab in combination with regorafenib (*n* = 2), lenvatinib followed by sorafenib (*n* = 1), linifanib followed by sorafenib (*n* = 1), nivolumab (*n* = 1), ramucirumab (*n* = 1)

### Algorithm performance

**Fig. 2:**
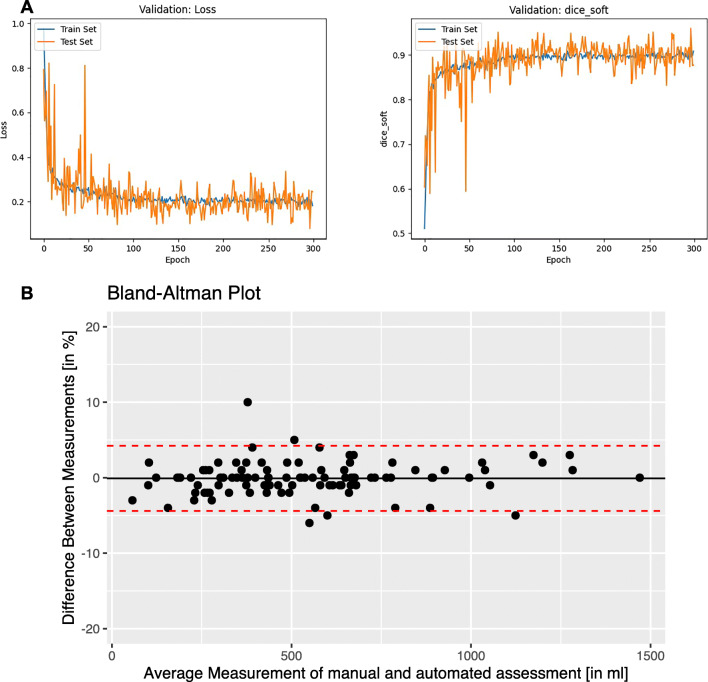
The course of training of the convolutional neural network (**A**) ((*left*) Tversky loss values over the number of epochs; (*right*) Sørensen Dice Scores over the number of epochs; train set: 70 sets of manually segmented spleen data; test set: 30 different sets of manually segmented spleen data); Bland-Altman Plot shows the distribution of manually and automatically assessed splenic volumes (**B**)

The manual assessments yielded a mean splenic volume of 549.7 ml for the training dataset and 524.3 ml for the validation dataset. The algorithm assessments yielded a mean splenic volume of 550.8 ml for the training set and 512.6 ml for the validation set. The mean difference between the manual and algorithm volume assessments was 0.1%, with a standard deviation of 2.2%. The training progress of the convolutional neural network is depicted in Fig. 2a; the distribution of volumes is shown in a Bland-Altman Plot in Fig. 2b. The Sørensen Dice coefficients were 0.96 for both the training and validation sets.

**Fig. 3:**
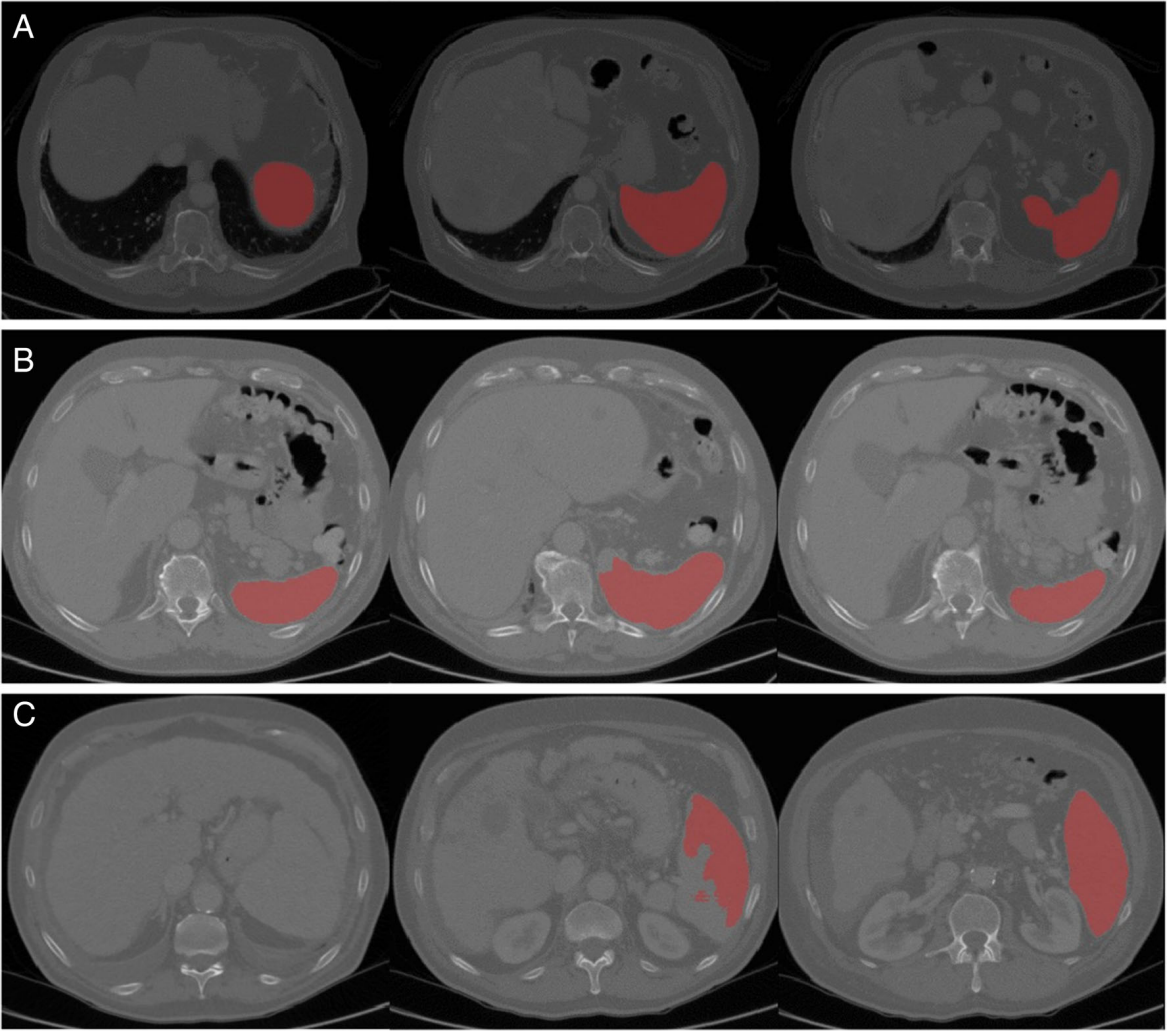
Representative images of the algorithm’s performance (from left to right images at upper, middle, and lower part of the spleen): **A** perfect segmentation, **B** acceptable segmentation (minor segmentation error medially), **C** poor segmentation (major segmentation error in the upper part with kissing liver and spleen phenomenon)

Next, we assessed the automated segmentation for the remaining patient cohort (*n* = 227). We found that 206 (90.7%) measurements were rated perfect, 17 (7.5%) were rated acceptable, and four (1.8%) were rated poor, after consensus reading. Representative images of each grade are shown in Fig. 3; the complete animation of each example is provided in the supplement (Supplementary Figs. S1a-S1c). For the four cases with poor ratings, we performed additional manual segmentations to obtain the proper splenic volume.

### Splenic volume

**Fig. 4:**
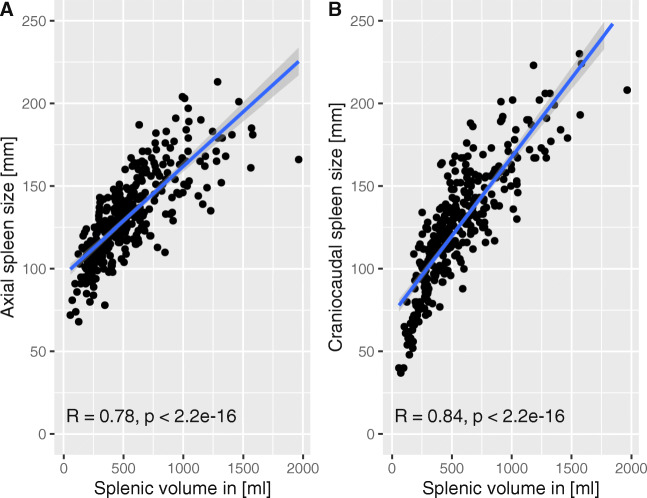
Correlation between two-dimensional splenic measurements and splenic volume. **A** Axial spleen size; **B** craniocaudal spleen size

The mean splenic volume for the whole patient cohort was 551.2 ml. In addition, the mean axial diameter was 132.3 mm and the mean craniocaudal diameter 125.0 mm. Both the axial and craniocaudal diameters were significantly correlated with the splenic volume (*p* < 0.001, Fig. 4), with high Pearson coefficient values (0.78 and 0.84, respectively).

### Survival analysis

**Fig. 5:**
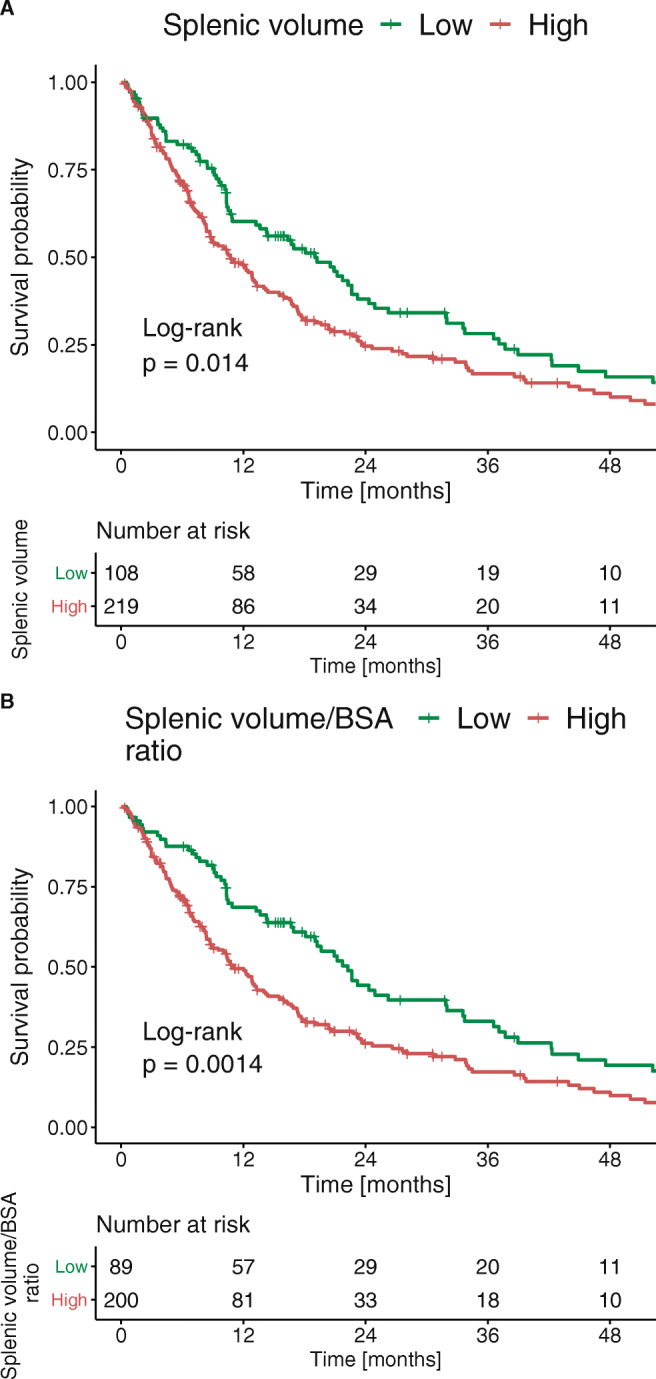
**A** Kaplan–Meier survival curves show survival of patients with low (green) and high (red) splenic volumes (*n* = 327); **B** Kaplan–Meier survival curves show survival of patients with low (green) and high (red) splenic volume-to-BSA ratio (*n* = 289)

Based on an optimal stratification of the median OS, we found that the best cut-off value for predicting mortality risk was a splenic volume of 382.6 ml. With this cut-off value, 219 (67.0%) patients had high splenic volumes and 108 (33.0%) had low splenic volumes. The median OS of patients with high and low splenic volumes were 10.6 and 18.8 months, respectively (*p* = 0.014, Fig. 5a).

The optimal stratifications of axial and craniocaudal spleen sizes yielded cut-off values of 163 and 115 mm, respectively. Neither index showed a significant association with OS in univariate analyses. The median OS times were 9.9 and 13.1 months (*p* = 0.220) for high and low axial spleen diameters, respectively (Supplementary Fig. S2A), and 11.9 and 16.1 months (*p* = 0.063), for high and low craniocaudal spleen diameters, respectively (Supplementary Fig. S2B).

A univariate Cox hazard regression analyses identified a high splenic volume, low albumin, high bilirubin, high AST, and a large tumor size as significant prognostic factors (Table 2). None of the other included risk factors showed a significant association with OS. In the subsequent multivariate analysis, which included all the significant abovementioned factors, splenic volume did not reach significance.

**Table 2:** Univariate and multivariate Cox regression results of factors related to survival for all patients (*n* = 327)

Covariate	Category	Univariate			Multivariate		
		HR	95% CI	*p*-value	HR	95% CI	*p*-value
Age	*≥ 70 years*	1.0	0.8–1.3	0.920			
AFP	*> 400 ng/ml*	1.0	0.7–1.2	0.770			
Albumin level	*≥ 35 g/l*	2.2	1.7–2.9	** < 0.001**	1.7	1.3–2.4	** < 0.001**
Bilirubin level	*≥ 1.2 mg/dl*	2.1	1.7–2.8	** < 0.001**	2.0	1.5–2.6	** < 0.001**
AST level	*> 31 U/l*	1.8	1.1–3.1	**0.033**	1.8	1.0–3.2	**0.047**
ALT level	*≥ 35 U/l*	1.2	0.9–1.6	0.190			
INR level	*> 1.2*	1.1	0.8–1.4	0.550			
Platelet count	*> 100 /nl*	1.0	0.8–1.3	0.850			
Tumor number	*≥ 2*	1.3	0.9–1.7	0.150			
Max. lesion size	*> 5.0 cm*	1.3	1.0–1.8	**0.037**	1.4	1.0–3.2	**0.028**
Splenic volume	*High*	1.4	1.1–1.8	**0.014**	1.1	0.9–1.5	0.354
Axial spleen size	*High*	1.2	0.9–1.8	0.220			
Craniocaudal spleen size	*High*	1.3	1.0–1.7	0.064			

Significant *p* values are marked in bold

*HR* hazard ratio, *CI* confidence interval, *AFP* alpha fetoprotein, *AST* aspartate aminotransferase, *ALT* alanine aminotransferase

Subsequently, we analyzed the role of the splenic volume normalized to BSA for all patients with available information on body weight and height (*n* = 289). Based on an optimal stratification of the median OS, we found that the best cut-off value for predicting mortality risk was a splenic volume-to-BSA ratio of 192.7 ml/m^2^. With this cut-off value, 200 (69.2%) patients had high splenic volume-to-BSA ratio, and 89 (30.8%) had low ratio. The median OS of patients with high and low splenic volume-to-BSA ratio were 10.9 and 22.0 months, respectively (*p* = 0.001, Fig. 5b).

A univariate Cox hazard regression analyses identified a high splenic volume-to-BSA ratio, low albumin, high bilirubin, and a large tumor size as significant prognostic factor (Table 3). None of the other included risk factors showed a significant association with OS. In multivariate analysis, splenic volume-to-BSA ratio remained an independent prognostic predictor, as did the other abovementioned significant factors.

**Table 3:** Univariate and multivariate Cox regression results of factors related to survival for patients with available body surface area (*n* = 289)

Covariate	Category	Univariate			Multivariate		
		HR	95% CI	*p*-value	HR	95% CI	*p*-value
Age	*≥ 70 years*	1.0	0.8–1.3	0.950			
AFP	*> 400 ng/ml*	1.0	0.7–1.2	0.700			
Albumin level	*≥ 35 g/l*	2.2	1.6–3.0	** < 0.001**	1.8	1.3–2.5	** < 0.001**
Bilirubin level	*≥ 1.2 mg/dl*	2.1	1.6–2.8	** < 0.001**	1.9	1.4–2.5	** < 0.001**
AST level	*> 31 U/l*	1.7	1.0–2.9	0.064			
ALT level	*≥ 35 U/l*	1.2	0.9–1.6	0.220			
INR level	*> 1.2*	1.1	0.8–1.5	0.650			
Platelet count	*>100*	1.0	0.8–1.3	0.910			
Tumor number	*≥ 2*	1.2	0.9–1.7	0.190			
Max. lesion size	*> 5.0 cm*	1.3	1.0–1.8	**0.045**	1.4	1.1–1.9	**0.017**
Splenic volume/BSA ratio	*High*	1.6	1.2–2.2	**0.002**	1.4	1.0–1.9	**0.046**

*HR* hazard ratio, *CI* confidence interval, *AFP* alpha fetoprotein, *AST* aspartate aminotransferase, *ALT* alanine aminotransferase, *BSA* body surface area

Furthermore, we analyzed TTP and TTUP of these patients with regard to the normalized splenic volume using the same cut-off value. The median TTP of patients with high and low splenic volume-to-BSA ratio were 6.7 and 11.1 months, respectively (*p* = 0.10, Supplementary Fig. S3A). The median TTUP of patients with high and low splenic volume-to-BSA ratio were 9.9 and 27.8 months, respectively (*p* < 0.001, Supplementary Fig. S3A).

### Influence on subsequent treatment

The median splenic volume of patients who were able to receive multiple TACE sessions was significantly lower (458 ml vs 582 ml, *p* < 0.001). Furthermore, the median splenic volume of patients who received subsequent treatment was also significantly lower (399 ml vs 520 ml, *p* < 0.001).

### Risk of hepatic decompensation

**Fig. 6:**
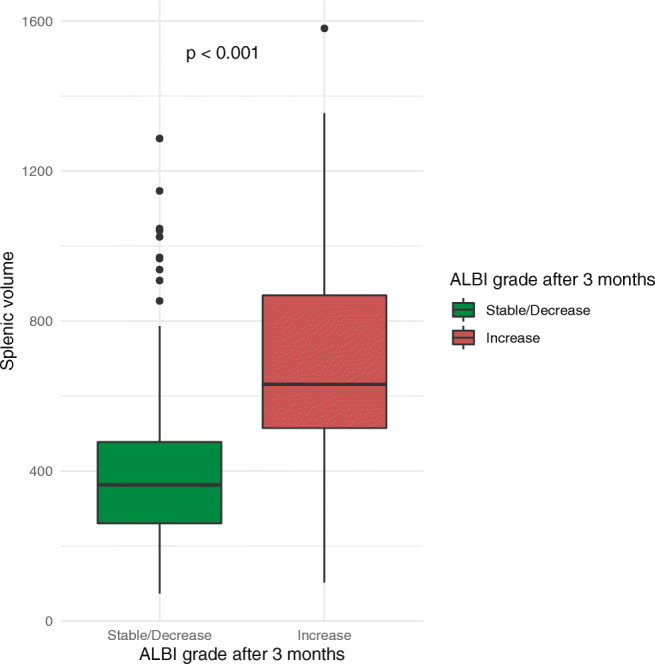
Boxplot showing the distribution of the splenic volume among patients with a stable/decrease ALBI grade (green) and patients with an increased ALBI grade (red) 3 months after TACE

We further assessed the risk of an increase in the ALBI grade for patients who had an initial grade of 1 or 2 and an available follow-up ALBI value 3 months after TACE (*n* = 197, 60.2%). Of these patients, a total of 61 (31.0%) patients had an ALBI grade increase 3 months after TACE. The median splenic volume of these patients was significantly higher (632 ml, IQR 514–868 ml) in comparison to patients without an increase (363 ml, IQR 261–477 ml) (*p* < 0.001, Fig. 6). Receiver operating characteristic analysis revealed an AUC of 0.83 with a sensitivity of 91.2% and a specificity of 72.1% at the cut-off of 455.3 ml for predicting an ALBI increase.

## Discussion

This study was the first to assess the prognostic role of splenic volume for patients with HCC undergoing TACE in a Western patient cohort. Here, we developed a fully automated approach, based on deep-learning methods, for assessing splenic volume.

Manual assessments of splenic volume are time-consuming, and they run a high risk of interrater variance [13]. Thus, we built a deep learning–based tool to assess splenic volume automatically, based on CT images. The U-Net architecture used for segmentation yielded a Sørensen Dice coefficient of 0.96 for training and 0.96 for validation. These coefficients indicated excellent algorithm performance. Additionally, the manual and automatic splenic volume assessments only differed by 0.1%.

The time consumption and technical challenges of manual splenic volume assessments [13] have hindered their integration into clinical workflows, despite reports that splenic volume was a highly predictive factor for several cancer entities, including HCC [9–11]. Historically, several surrogates have been proposed for rapid estimations of splenic volume. For patients with liver cirrhosis, the axial and craniocaudal diameters of the spleen have been identified as precise surrogates of splenic volume [13]. Our results also indicated that these diameters were moderately to highly correlated with splenic volume. However, neither the craniocaudal nor the axial diameter was a relevant prognostic factor, because neither reached significance, even with optimal stratification, in our cohort. Thus, when deciding whether to use estimates for spleen size or true splenic volume for assessing risk in patients with HCC undergoing TACE, true splenic volume should be favored.

AI-based algorithms can potentially simplify the radiologist’s work in daily clinical routines [14]. Tasks that can be readily simplified and automized include organ segmentation, volume assessments, and body composition assessments [29–31]. Recently, deep learning algorithms have also been used for the assessment of splenic volume in the context of variceal detection [32]. AI-based algorithms have the advantage of being easy to integrate into clinical workflows and automated quantitative reports can be automatically sent to the local image archiving and communication system. However, currently, those new technologies require evaluation in the context of clinical applications. Accordingly, specific use cases are mandatory.

While there is an initial threshold to install and train a segmentation tool, which includes a manual one-time labelling of a training dataset, this effort is reduced thanks to publicly available software programs and libraries. Using other software for segmentation and U-Nets than the ones used in this study would have likely produced similar effective results, and once trained, no further user segmentation is needed to get an accurate splenic volume.

The literature is scarce regarding the prognostic role of splenic volume for patients with HCC undergoing TACE. To date, only one recent study by Dai et al showed that splenic volume was correlated to the Child-Pugh classification and OS [12]. The mean splenic volume of the 67 patients in that study was 300 ml, prior to TACE. That value was considerably lower than the mean volume of 551 ml in our patient cohort. In contrast to our study, the underlying etiology in all their patients was hepatitis B virus (HBV) infection. Unfortunately, they did not provide the number of patients with underlying cirrhosis. In general, most patients with chronic HBV infections and HCC do not have underlying cirrhosis. Thus, those patients are at lower risk of developing signs of portal hypertension, like an increased splenic volume. Accordingly, in that study, a smaller proportion of patients were in the high Child-Pugh class, compared to our cohort. Thus, those patients had better average liver function than the patients included in our study. All these factors might have explained the higher splenic volume in our patient cohort. Nevertheless, the two studies reported similar optimal cut-off values (373 ml vs 383 ml in our study) for high and low splenic volume.

Splenic volume was also significantly associated with both progression-free survival as well as hepatic decompensation and the likeliness to receive subsequent systemic treatment after TACE failure in our cohort. This is in line with prior findings that progression-free survival in TACE patients is linked to portal hypertension [33]. Moreover, prior studies have linked repeated TACE to an increase in portal hypertension [34] and have described the ALBI score as a predictor for failure of sorafenib treatment [35, 36].

In our study, we found that splenic volume prior to TACE has a high sensitivity of identifying patients with a post-treatment ALBI increase. Therefore, our study is the first identifying splenic volume as relevant prognostic imaging marker for hepatic decompensation in patients with unresectable HCC. In the context of emerging novel treatment options for patients with unresectable HCC, the optimal time-point for a treatment switch in the concept of stage migration is hard to identify [37]. However, a treatment switch is of utmost importance for the outcome of the patients as “an inappropriately high number of TACE sessions delays the switch to systemic therapy and may, in some cases, completely hinder the treatment switch due to the deterioration of liver function” [38]. Thus, splenic volume might function as an additional, currently underused parameter to identify patients at high risk for hepatic decompensation and therefore might lead to a tighter follow-up scheme and more frequent interdisciplinary discussion of these patients. However, no standard reference values neither for impaired survival nor increased risk of hepatic decompensation are currently available. Thus, future large-scale multicentric evaluation studies are needed to determine a generalizable cut-off value.

The present study had several limitations. First, it was a single-center, retrospective study. However, the sample size was distinctly larger than that included in the previous study on this topic [12]. Additionally, our dataset was well investigated and we only included patients with complete clinical, laboratory, and imaging data. Furthermore, missing values were not imputed. To avoid a time bias, we actively decided to include only patients from 2010 and later. These criteria minimized differences in the diagnosis and treatment decisions, which provided a more homogeneous study cohort. Furthermore, we excluded patients that underwent previous treatments to avoid other biases. Second, we included patients that underwent either conventional or drug-eluting bead-delivered TACE. However, several previous studies have shown that the TACE delivery technique did not influence the OS [39–41]. Third, we only used an internal validation set to assess algorithm performance. In the final prediction for the whole dataset, the neural network failed to provide an accurate prediction of splenic volume in four patients (1.8%). We restricted the training and validation cohort to 100 patients, determined a priori, to limit the burden of manual segmentation. Nevertheless, the neural network facilitated correct splenic volume calculations in 98.2% of non-segmented spleens. Therefore, the evaluation of this use case was not substantially hindered by the need to perform additional manual segmentations of those four spleens with grotesque anatomies. Consequently, we encourage future studies to employ neural networks for segmentation in validating the prognostic role of splenic volume for patients with HCC undergoing TACE.

In summary, we showed that training a deep learning algorithm was feasible for allowing fully automated splenic volume assessments for patients with HCC undergoing TACE. Compared to established two-dimensional estimates of splenic volume, our algorithm provided precise splenic volume assessments, which showed superiority in predicting survival and high sensitivity in identifying patients with a risk of hepatic decompensation. Thus, true splenic volume could serve as an additional imaging biomarker, available fully automatically without additional effort for every CT study.

## Supplementary Information

ESM 1 (DOCX 23299 kb)

## Abbreviations

AFP: Alpha fetoprotein
AST: Aspartate aminotransferase
BSA: Body surface area
CIs: Confidence intervals
CT: Computed tomography
HBV: Hepatitis B virus
HCC: Hepatocellular carcinoma
HRs: Hazard ratios
OS: Overall survival
TACE: Transarterial chemoembolization

## Declarations

### Guarantor

The scientific guarantor of this publication is PD Dr. med. Roman Kloeckner.

### Statistics and biometry

One of the authors has significant statistical expertise.

### Methodology

• retrospective

• diagnostic or prognostic study

• performed at one institution
